# Cardiovascular implications of COVID-19 versus influenza infection: a review

**DOI:** 10.1186/s12916-020-01816-2

**Published:** 2020-12-18

**Authors:** Muhammad Shahzeb Khan, Izza Shahid, Stefan D. Anker, Scott D. Solomon, Orly Vardeny, Erin D. Michos, Gregg C. Fonarow, Javed Butler

**Affiliations:** 1 Department of Medicine, Cook County Health Sciences, Chicago, IL USA; 2 Department of Medicine, Ziauddin Medical University, Karachi, Pakistan; 3 Department of Cardiology (CVK), and Berlin Institute of Health Center for Regenerative Therapies (BCRT), German Centre for Cardiovascular Research (DZHK) partner site Berlin, Charité Universitätsmedizin Berlin, Berlin, Germany; 4 Brigham and Women’s Hospital, Heart & Vascular Center, Boston, MA USA; 5 University of Minnesota, Minneapolis, MN USA; 6 Division of Cardiology, Johns Hopkins University School of Medicine, Baltimore, MD USA; 7 Division of Cardiology, Ronald Reagan-UCLA Medical Center, Los Angeles, CA USA; 8 Department of Medicine, University of Mississippi Medical Center, 2500 N. State Street, Jackson, MS 39216 USA

## Abstract

**Background:**

Due to the overlapping clinical features of coronavirus disease 2019 (COVID-19) and influenza, parallels are often drawn between the two diseases. Patients with pre-existing cardiovascular diseases (CVD) are at a higher risk for severe manifestations of both illnesses. Considering the high transmission rate of COVID-19 and with the seasonal influenza approaching in late 2020, the dual epidemics of COVID-19 and influenza pose serious cardiovascular implications. This review highlights the similarities and differences between influenza and COVID-19 and the potential risks associated with coincident pandemics.

**Main body:**

COVID-19 has a higher mortality compared to influenza with case fatality rate almost 15 times more than that of influenza. Additionally, a significantly increased risk of adverse outcomes has been noted in patients with CVD, with ~ 15 to 70% of COVID-19 related deaths having an underlying CVD. The critical care need have ranged from 5 to 79% of patients hospitalized due to COVID-19, a proportion substantially higher than with influenza. Similarly, the frequency of vascular thrombosis including deep venous thrombosis and pulmonary embolism is markedly higher in COVID-19 patients compared with influenza in which vascular complications are rarely seen. Unexpectedly, while peak influenza season is associated with increased cardiovascular hospitalizations, a decrease of ~ 50% in cardiovascular hospitalizations has been observed since the first diagnosed case of COVID-19, owing in part to deferred care.

**Conclusion:**

In the coming months, increasing efforts towards evaluating new interventions will be vital to curb COVID-19, especially as peak influenza season approaches. Currently, not enough data exist regarding co-infection of COVID-19 with influenza or how it would progress clinically, though it may cause a significant burden on an already struggling health care system. Until an effective COVID-19 vaccination is available, high coverage of influenza vaccination should be of utmost priority.

## Background

Coronavirus disease 2019 (COVID-19), caused by the severe acute respiratory syndrome coronavirus 2 (SARS-CoV-2), was first identified in December 2019 and has since evolved into a worldwide pandemic [1]. By September 2020, there have been over 25 million reported cases with over a million fatalities due to COVID-19 across 188 countries worldwide [2]. This has led to a major shift in reallocation of healthcare resources to cater the surge of COVID-19 patients by increasing inpatient beds dedicated to COVID-19 unit, expanding intensive care unit (ICU), and conducting large-scale testing for COVID-19. Increasing use of telemedicine, deferral of elective procedures and/or routine examinations, and physical distancing are also being adopted to minimize transmission rates [3].

Both SARS-CoV-2 and influenza virus share a variety of common features, including route of transmission and similar clinical presentations. A large proportion of COVID-19 patients have been reported to have pre-existing cardiovascular disease (CVD) which has been associated with worse prognosis [4–6]. It has also been suggested that COVID-19 may cause or precipitate myocardial injury and/or myocarditis and worsen heart failure due to a cytokine storm-related hyper-inflammation syndrome. Similar to SARS-CoV-2, influenza virus also has extensive effects on inflammatory [7] and coagulation pathways and is a well-known trigger for cardiovascular diseases [7, 8]. In addition to the underlying hyperinflammatory syndrome which is most commonly implicated in cardiovascular complications in both viruses, a substantial component of susceptibility is attributed to host genetics such as host frailty [9]. Although mortality comparisons between influenza and COVID-19 have been widely drawn, the mortality statistics used to compare the two have been scrutinized [10]. Recent studies suggest that SARS-CoV-2 is more lethal than prior respiratory infections, with a more potent inflammatory response that can possibly trigger more cardiovascular complications [11]. Influenza commonly peaks between December and February [12]. With projections estimating the COVID-19 outbreak to last for another year [13] and influenza season approaching, it is crucial to evaluate the differences in progression of the two diseases in patients prone to cardiovascular complications. In this review, we compare the effects of the two viruses on the cardiovascular system by focusing on the risk factors, short- and long-term complications, and mortality of both influenza and SARS-CoV-2.

## Main Text

PubMed and Scopus were searched to identify relevant studies using the following search terms: [(influenza OR flu) OR (Coronavirus disease 2019 OR COVID-19 OR severe acute respiratory syndrome coronavirus 2 OR SARS-CoV-2)] AND (cardiovascular disease OR cerebrovascular disease OR cardiac abnormality OR cardiac injury OR mortality OR cardiovascular mortality OR cardiac failure OR vascular disease OR stroke OR heart failure OR myocardial infarction OR MI OR ischemic heart disease OR acute coronary syndrome OR myocarditis OR cardiac biomarker OR troponin OR creatine kinase OR thromboembolism OR thrombosis OR vaccine OR prevention). Studies were included if they (1) were pertaining acute (1–7 days since diagnosis) or chronic influenza (> 2 weeks since diagnosis) with concurrent cardiac abnormality, (2) evaluated COVID-19 with concomitant cardiac dysfunction upon presentation or during hospitalization, (3) evaluated short- and/or long-term complications, (4) diagnosed influenza via influenza-like symptoms or serology, and (5) were original studies including observational studies, time-series analysis, case-cross over studies, or meta-analysis. Study designs including case-reports, short case series, commentaries, letter to editors, and editorials were excluded. Main outcomes of interest included (1) mortality, (2) ICU admissions, (3) cardiac biomarkers, (4) vascular complications, (5) cardiac events, (6) stroke, (7) cardiovascular hospitalizations, and (8) prevention strategies. References of relevant review articles were also hand-searched to ensure no relevant studies were missed.

### Cardiovascular mortality rates with influenza and COVID-19

**Fig. 1:**
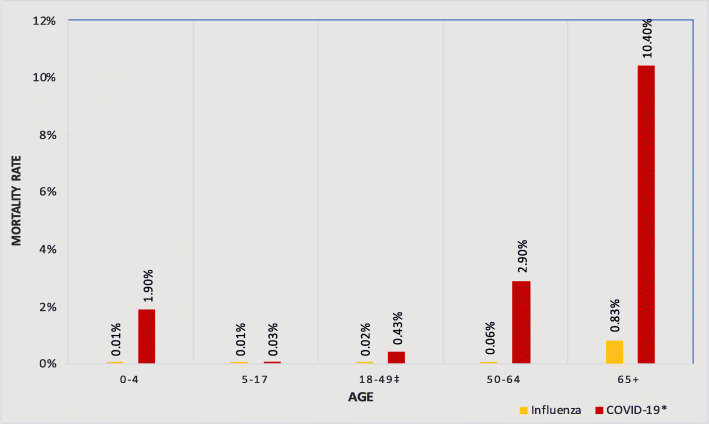
Comparison of estimated influenza vs confirmed COVID-19 death rate by age. *CDC confirmed COVID-19 death rate as of June 23, 2020. ^‡^Average of COVID-19 death rate obtained for age group 18–29, 30–39, and 40–49

Comparing mortality rates of influenza and COVID-19 is challenging owing to the variances in data accounting for the two diseases. Adult influenza is usually a self-limiting disease; hence, it is often not reported to the public health authorities subsequently leading to under-reporting of data. Similarly, lack of testing also poses limitations in reporting of COVID-19 and exact epidemiology of asymptomatic or mild non-specific symptoms related to COVID-19 is not well characterized. Mortality risk comparison between the two is complicated by the adoption of different metrics in comparing mortality statistics between the two diseases [10]. The mortality rate of influenza is most commonly reported as the estimated seasonal influenza deaths reported annually by the Centers for Disease Control and Prevention (CDC) in contrast to raw counts of mortality reported directly for COVID-19 [10] (Fig. 1). This may lead to inaccurate conclusion when directly comparing the data. Although limited by failure to account for missing data and yielding premature results, case fatality ratio is being increasingly utilized to highlight the burden of disease in a given population. The overall case fatality ratio of COVID-19 across China after adjusting for censoring, demography, and under-ascertainment has been reported to be 1.38% (95% CI [1.23–1.53]) [14] which is almost 15 times higher than that of seasonal influenza (0.0962%) [15, 16].

Ten observational studies have reported mortality data of acute influenza concurrent with cardiac abnormality upon presentation. Mortality in individuals hospitalized due to influenza or influenza-like illness ranged from 3.8 to 50% in these studies, with the exception of one study by Chacko et al. [17] evaluating cardiac manifestations in patients with severe H1N1 virus during the 2009 pandemic, where a 92% overall mortality rate was observed (Table 1). In contrast to acute influenza, over the course of last 5 months, many (*n* = 16) observational studies have reported mortality data for patients with COVID-19 infection. The overall mortality in this patient cohort has ranged from 1.4 to 61.5%, which is higher than that observed in patients with acute influenza (Table 1).

**Table 1:** Major observational studies of acute influenza and COVID-19 concurrent with cardiac abnormality and mortality

Study	Country	Design	(***N***)	HTN (%)	DM (%)	CVD (%)	EF < 50 (%)	Influenza and cardiac abnormality (%)	Mortality, ***N*** (%)	Mortality and cardiac events
**Acute influenza**										
Chacko et al. [17]	India	Retrospective	37	NR	5.4	5.4	54.0	80.8	34 (91.9)	Crude mortality rate 93% with myocarditis vs 38% without myocarditis
Fagnoul et al. [18]	Belgium	Retrospective	46	NR	NR	10.9	NR	60.9	23 (50.0)	Mortality reported similar between patients with and without pre-existing CVD
Han et al. [19]	China	Retrospective	40	52.5	NR	0.2	2.5	55.0	5 (12.5)	NR
Ludwig et al. [20]	USA	Retrospective	600	89.5	46.9	28.8	NR	23.8	18/143 (12.6)	Eleven (61%) of those who died received a diagnosis of NSTEMI or probable NSTEMI ≤ 30 days after laboratory-confirmed influenza virus specimen collection.
Harris, 2019 [21]	USA	Retrospective	33	NR	NR	42.4	36.4	100	4 (12.1)	All patients who expired while inpatient had no previous documented cardiac history.
Panhwar, 2019 [22]	USA	Retrospective	54,590	75.0	33.1	NR	100	100	3439* (6.3)	NR
Vejpongsa, 2019 [23]	USA	Prospective	1,863,615	73.2	46.0	0.5^‡^	0.3	0.5	1305/9885 (13.2)	NR
Panhwar, 2019 [24]	USA	Retrospective	45,460	NR	NR	NR	100	100	2818* (6.2)	NR
Pizzini et al. [25]	Austria	Cross-sectional analysis	264	NR	NR	33.7	NR	31.8	10 (3.8)	Higher high-sensitivity cardiac troponin T levels were observed in patients who died within 30 days when compared to patients who survived
Gao et al. [26]	China	Retrospective, Cohort	321	NR	13.1	8.1	34.6	63.2	154 (48.0)	130 patients who died had cardiac injury vs 24 patients who did not have cardiac injury
**Summary Estimate, % (95% CI)**^§^	NA	NA	111,276^$^	74.5 (71.8, 77.1)	30.4 (22.7, 39.4)	11.4 (1.5, 52.4)	13.0 (0.8, 73.5)^#^	87.8 (43.8, 98.5)	17.0 (12.3, 23.0)	NA
**COVID-19**										
Cummings et al. [27]	USA	Prospective Cohort	257	63.0	35.8	19.1	NR		101 (39.3)	Older age, chronic cardiac disease, chronic pulmonary disease, higher concentrations of IL-6, and higher concentrations of D-dimer were independently associated with in-hospital mortality.^~^
Chen et al. [28]	China	Retrospective	99	NR	NR	40.4	NR		11 (11.0)	NR
Richardson et al. [29]	USA	Retrospective case-series	5700	53.1	31.7	16.9	6.5		553 (9.7)	Mortality was 0% for male and female patients younger than 20 years. Mortality rates were higher for male compared with female patients at every 10-year age interval older than 20 years.^~^
Goyal, 2020 [30]	USA	Retrospective case-series	393	50.1	25.2	13.7	NR		40 (10.2)	NR
Arentz et al. [31]	USA	Retrospective case-series	21	NR	33.3	NR	42.9		11 (52.4)	NR
Zhou et al. [32]	China	Retrospective Cohort	191	30.4	18.8	7.9	NR		54 (28.3)	Odds of in-hospital death was higher in patients with diabetes or coronary heart disease. Age, lymphopenia, leukocytosis, and elevated ALT, lactate dehydrogenase, high-sensitivity cardiac troponin I, creatine kinase, d-dimer, serum ferritin, IL-6, prothrombin time, creatinine, and procalcitonin were also associated with death.^~^
Huang et al. [4]	China	Cohort	41	14.6	19.5	14.6	NR		6 (14.6)	NR
Guan et al. [33]	China	Retrospective	1099	15.0	7.4	2.5	NR		15 (1.4)	NR
Wang et al. [34, 35]	China	Retrospective case-series	138	31.2	10.1	14.5	NR		6 (4.3)	NR
Guo et al. [5]	China	Retrospective	187	32.6	15.0	15.5	NR		43 (23.0)	NR
Yang et al. [36]	China	Retrospective	52	NR	17.3	10.0	NR		32 (61.5)	NR
Wu et al. [37]	China	Cohort	201	19.4	10.9	4.0	NR		44 (21.9)	Patients who died were older and had higher proportions of hypertension.^~^
Chen et al. [38]	China	Retrospective	274	33.9	17.2	8.4	0.4		113 (41.2)	NR
Wang et al. [34]	China	Retrospective	339	40.8	15.9	15.7	NR		65 (19.2)	Older age was shown to increase the likelihood of death in elderly patients. Comorbidities including cardiovascular disease cerebrovascular disease were all predictive of fatal outcomes.^~^ Complications including acute cardiac injury, arrhythmia, acute kidney injury, ARDS, cardiac insufficiency, and bacterial infection were all predictors of death.^~^
Shi et al. [39]	China	Cohort	416	30.5	14.4	10.6	NR		57 (13.7)	A significantly higher risk of death was observed in patients with cardiac injury than in those without cardiac injury.^~^
Yu et al. [40]	China	Prospective	226	42.5	20.8	11.5	1.8		9 (4.0)	NR
**Summary estimate, % (95% CI)**^§^	NA	NA	9634	34.3 (25.7, 44.0)	18.2 (13.4, 24.1)	11.9 (9.0, 15.7)	4.9 (1.1, 19.3)		16.8 (11.1, 24.8)	NA

*NR* not reported, *NA* not applicable, *HTN* hypertension, *DM* diabetes mellitus, *CVD* cardiovascular disease, *EF* ejection fraction, *ARDS* acute respiratory distress syndrome

^‡^Calculated by aggregating prior MI, prior CV surgery, CHF, and VHD

*Incidence before propensity matching (influenza + HF)

^~^Results based on the analysis of Cox regression

^§^Summary estimates were calculated using meta-analysis of proportions. Logit transformed proportions and corresponding 95% confidence intervals (CIs) from studies reporting the aforementioned data were pooled using random-effects model and presented as % (95% CI)

^$^Popoled estimate includes 9885 patients diagnosed with acute influenza from Vejpongsa study.

^#^Since both Panhwar 2019 studies report influenza in HF patients, they were not included in the pooled analysis

Pooled proportional analysis of mortality excluding Chacko 2012 was 13.8% (9.9%, 19.0%)

A correlation between cardiovascular mortality and influenza has been suggested by various epidemiological studies. In time-series analyses, an increased mortality due to ischemic heart disease and acute myocardial infarction (MI) has been observed to coincide with peak influenza seasons [8, 41, 42]. Kwong et al. found there was a 6-fold increased risk of MI within 7 days of confirmed influenza diagnosis, compared to a control period [43]. Madjid et al. studied 34,892 autopsy findings during influenza epidemics over 7 years and concluded there was an increased odds of death due to acute MI (OR 1.30 [1.08–1.56]) during influenza epidemic season compared to off-season [8]. Nguyen et al. demonstrated a correlation between seasonal average influenza incidence and excess cardiovascular mortality (Pearson correlation coefficients ≥ 0.75, *P* ≤ .05 for 4 different influenza indicators) among adults 65 years and older [41]. Although studies pertaining to acute infection and subsequent mortality are a better indication of a correlation between the disease and mortality, these data are limited by complications which may occur in patients once discharged from hospital and is also difficult to obtain in patients with acute influenza, given the vast under-reporting of the disease.

Given that no time-series analysis pertaining to COVID-19 exists yet, a comparison between both diseases and cardiovascular mortality can be best appreciated by observational studies accounting for the given data. Four influenza studies have reported increased mortality in patients with acute influenza and concomitant cardiac injury. Both Pizzini et al. [25] and Gao et al. [26] reported elevated cardiac biomarkers reflective of myocardial injury (cardiac troponin) upon hospitalization to be associated with increased acute cardiac events and mortality. Death due to myocarditis is also common among patients presenting with influenza. In a retrospective study of 74 patients, To et al. reported myocarditis as the cause of death in 22% of fatal cases [44]. Similar to influenza, increased cardiovascular mortality has been observed in patients with COVID-19. The magnitude of increased risk of cardiovascular mortality with COVID-19 can be best inferred by Zhou et al. [32] study, where non-survivors showed higher rates of acute cardiac injury compared with survivors (59% vs 1%), and study by Guo et al. [5] where highest mortality rate (69.4%) was observed in patients with elevated troponin T and underlying CVD, followed by patients with elevated troponin T and no underlying CVD (37.5%). In another study from Wuhan, China, Shi et al. found that the presence of an elevated high-sensitivity cardiac troponin I was associated with a 4-fold increased risk of death even after adjusting for age and pre-existing CVD [39].

An association between underlying risk factors, particularly CVD and mortality due to influenza, has been less commonly reported. Two studies [18, 21] have reported no association between mortality and pre-existing CVD, thereby indicating no significance of cardiac history in the exacerbation of disease. This contrasts with data observed in COVID-19 infection, where patients with cardiovascular risk factors appear to be at an increased risk for fatal outcomes associated with COVID-19 disease [5, 32, 34, 39]. About 15 to 70% of COVID-19-related reported deaths have had underlying CVD [5, 32, 34, 38]. Given the adverse outcomes in this patient cohort, this association warrants further research to determine if COVID-19 has a specific predisposition for patients with pre-existing CVD.

### Intensive care unit (ICU) admission between influenza and COVID-19

Limited data exist for acute influenza with concurrent cardiac abnormality and subsequent ICU admission. Of the six studies [17–20, 26, 45] which have reported ICU admissions for patients with influenza, four studies [17–19, 45] evaluated clinical progression in patients directly admitted to the ICU. Of the remaining two studies, ICU admissions accorded for 1% and 81% (reporting clinically severe patients during H1N1 pandemic) of all patients hospitalized due to acute influenza [20, 26] (Table 2). Of all the studies reporting data, median number of ICU stay ranged from 6 to 12 days with 57 to 68% patients requiring invasive mechanical ventilation. Underlying CVD was the most common comorbidity associated with patients requiring ICU care, with pre-existing CVD ranging from 5.4 to 35.2% in this patient cohort [17–20, 45].

**Table 2:** Major observational studies of acute influenza and COVID-19 reporting ICU admission

Study	Country	Design	(***N***)	Pre-existing CVD for patients in ICU, (%)	ICU, ***N*** (%)	ICU stay, days	Requiring invasive mechanical ventilation, ***N*** (%)
**Acute influenza**							
Chacko et al. [17]	India	Retrospective	37	5.4	37 (100)	12 (9–15)	25 (67.6)
Fagnoul et al. [18]	Belgium	Retrospective	46	10.9	46 (100)	9 (2–16)	26 (56.5)
Han et al. [19]	China	Retrospective	40	15.0	40 (100)	NR	NR
Ludwig et al. [20]	USA	Retrospective	600	28.8	44/143 (30.8)	6 (2–30)	NR
Chao et al. [45]	Taiwan	Retrospective	125	35.2	125 (100)	12.7 (10.2)*	NR
Gao et al. [26]	China	Retrospective	321	NR	260 (81.o)	10 (3–20)	196 (61.1)
**Summary estimate, % (95% CI)**^‡^	NA	NA	1169	20.6 (13.2, 30.7)	93.8 (74.7, 98.7)^§^	NA	61.1 (56.3, 65.7)
**COVID-19**							
Cummings et al. [27]	USA	Prospective Cohort	257	NR	203 (79.0)	NR	203 (80.0)
Richardson et al. [29]	USA	Retrospective case-series	5700	NR	1281 (22.5)	NR	1151 (20.2)
Goyal, 2020 [30]	USA	Retrospective case-series	393	NR	NR	NR	130 (33.1)
Arentz et al. [31]	USA	Retrospective case-series	21	NR	21 (100)	NR	NR
Chen et al. [28]	China	Retrospective	99	NR	NR	NR	4 (4.0)
Zhou et al. [32]	China	Retrospective Cohort	191	NR	50 (26.2)	8 (4–12)	32 (16.8)
Huang et al. [4]	China	Cohort	41	NR	13 (31.7)	NR	2 (4.9)
Guan et al. [33]	China	Cohort	1099	NR	55 (5.0)	NR	25 (2.3)
Wang et al. [35]	China	Retrospective case-series	138	8.0	36 (26.1)	NR	6 (4.3)
Yang et al. [36]	China	Retrospective	52	9.6	52 (100)	NR	22 (42.3)
Wu et al. [37]	China	Cohort	201	NR	53 (26.4)	NR	5 (2.5)
Shi et al. [39]	China	Cohort	416	NR	NR	NR	32 (7.7)
Yu et al. [40]	China	Prospective	226	11.5	226 (100)	NR	85 (37.6)
**Summary estimate, % (95% CI)**^‡^	NA	NA	8834	10.2 (7.6, 13.5)	47.2 (28.9, 66.3)	NA	14.4 (8.0, 24.5)

ICU stay reported as median (IQR)

*ICU* intensive care unit, *CVD* cardiovascular disease, *NR* not reported, *NA* not applicable

*Data reported as mean (SD)

^‡^Summary estimates were calculated using meta-analysis of proportions. Logit transformed proportions and corresponding 95% confidence intervals (CIs) from studies reporting the aforementioned data were pooled using random-effects model and presented as % (95% CI)

^§^Results including studies presenting 100% ICU admissions. Pooled proportional analysis of Gao 2020 and Ludwig 2017 yielded 58.0% (13.1%, 92.7%)

In contrast to influenza, many studies have reported data for ICU admissions of patients hospitalized due to COVID-19 infection [4, 27, 29, 31–33, 35–37, 39, 40]. The ICU admissions have ranged from 5 to 79% of all patients hospitalized due COVID-19 (Table 2) [4, 27, 29, 32–34, 37]. Aggregate data from Italy showed that 12% of all COVID-19 hospitalized patients required ICU admission [46, 47]. This is higher than the expected rates of ICU admission for patients due to influenza, whereby approximately 10% of those hospitalized due to severe respiratory distress may require ICU admission [48]. These numbers should be interpreted with caution given that an increasingly higher number of COVID-19 positive patients require hospitalization in a very short time when compared to influenza for whom hospitalization counts are usually calculated over a longer time period. For example, Taylor et al. conducted a study to identify mortality of patients admitted in the ICU due to influenza [49]. Over a period of 6 years, patients admitted to ICU due to severe influenza accounted for only 17.9% of all hospitalized influenza patients [49], thereby indicating that the severity of severe influenza may not be an imminent threat to healthcare resources, unlike COVID-19 which is exerting significant burden on the current critical care units. This can be further substantiated by a multicenter study including 326 patients (211 COVID-19; 115 influenza) where none of the influenza patients required ICU admission compared with COVID-19 patients, where 21.3% patients required ICU care [50].

Despite an increased plausible association between cardiovascular comorbidity and increased severity of SARS-CoV-2 [6, 32], only three studies have evaluated pre-existing CVD for critically ill patients in the ICU. Acute respiratory distress syndrome (ARDS) was the most common complication (60–70%) in patients admitted to ICU, followed by shock (30%), myocardial dysfunction (20–30%) and acute kidney injury (10–30%) [4, 35, 36, 51]. Of all the studies reporting data, approximately 3 to 80% patients required invasive mechanical ventilation. One major complication of both viral infections is the immune susceptibility to secondary bacterial superinfection, which can often lead to adverse outcomes despite initial improvement. Nosocomial infections including ventilator-associated infections are often unavoidable in ICU patients, particularly during a pandemic [52]. During the 2009 H1N1 influenza pandemic, up to 34% of all deaths were due to bacterial co-infections [53]. This highlights the burden of increasing resources needed to curtail this pandemic.

### Cardiac biomarkers in influenza and COVID-19

Heterogeneous data exist for cardiac biomarkers evaluating the risk of myocardial injury in patients presenting with influenza (Additional file 1: Table S1A). Eleven studies have reported at least one cardiac biomarker for patients presenting with acute influenza [17–19, 25, 26, 54–58]. Ludwig et al. [20] showed that among 600 veterans with influenza who had a cardiac biomarker test performed ≤ 30 days after the influenza laboratory test, 143 had a troponin or CK-MB > 99% of the upper reference limit. All these patients had at least one risk factor for CVD, with older veterans with influenza and cardiac injury being more susceptible to cardiac complications at the time of influenza diagnosis [20]. Greaves et al. [58] and Ison et al. [56] reported elevated creatine kinase levels (CK) upon enrollment [56, 58]. Although there are now highly sensitive troponin assays which are more sensitive for detecting cardiac injury compared to conventional troponin assays [59, 60], only one study evaluated high-sensitivity cardiac troponin T (hs-cTnT) for cardiac injury risk among patients presenting with influenza [25]. Similarly, only one study was found which evaluated D-dimer levels upon hospitalization in patients with acute influenza and myocardial injury [61].

In contrast to influenza, less heterogeneity was noted in cardiac biomarkers evaluating risk of myocardial injury in patients with COVID-19, with fourteen studies reporting at least two cardiac biomarkers for patients hospitalized due to COVID-19 [4, 5, 27, 29, 31–39, 62, 63]. Majority of the studies evaluated hs-cTnT and CK upon hospitalization and included results for D-dimer values (Additional file 1: Table S1B). An elevated D-dimer value has been independently associated with increased in-hospital mortality in patients with COVID-19 [64]. Analysis of the initial studies from China shows a considerable proportion of patients (12–28%) to have elevated cardiac troponin upon hospitalization [4, 5, 32, 36]. This proportion is much higher than that observed in patients presenting with influenza and myocardial injury, thereby indicating a much potent inflammatory response of SARS-CoV-2. In addition to cardiac biomarkers, inflammatory biomarkers such as C-reactive protein, IL-6, and ferritin also provide useful information regarding the underlying hyperinflammatory state of the patient. In SARS-CoV-2, there is an initial phase of disease followed by, in minority of cases, by worsening of the disease that is not related to the viral growth but to the underlying hyperinflammatory state which is less commonly observed in influenza. The timing and subsequent elevation of these aforementioned biomarkers therefore relays the prognostic information of the course of the disease, especially during the first 7–10 days where an increase in these biomarkers may help clinicians to evaluate the severity of the disease. Therefore, for patients presenting with mild increase in cardiac troponin upon hospitalization which may decrease over the course of disease, the cardiac biomarkers reflect an underlying inflammatory response which may resolve over time. In patients who present with increase in cardiac biomarkers during the course of the disease however, the increase may indicate the adverse progression of cytokine-mediated endothelial injury and an unfavorable prognosis. Of note, patients presenting with elevated troponin levels upon admission show higher in-hospital mortality [5, 39]. This was evaluated in a meta-analysis of four studies as well, where the value of troponin I was significantly higher in patients with severe COVID-19 (standardized mean difference (SMD), 25.6 ng/L; 95% CI, 6.8–44.5 ng/L; *I*^2^ = 98%) compared to those with the milder form of disease [65]. Similar association using robust analysis is yet to be conducted for studies evaluating troponin levels and mortality in patients with influenza.

### Vascular complications in influenza and COVID-19

A markedly increased incidence of intravascular complications, particularly venous and arterial thromboembolic disease, has been reported in patients with severe SARS-CoV-2. In a study by Klok et al., despite systemic thromboprophylaxis, up to 31% patients developed thrombotic complications, with pulmonary embolism (PE) accounting for the majority of complications [62]. Frequent venous and arterial thrombotic events have been reported in other studies as well, with rates ranging from 27 to 69% for peripheral venous thromboembolism (VTE) and up to 79% for deep vein thrombosis and PE [66–68]. COVID-19 may predispose patients to intravascular thrombosis due to excessive inflammation increasing the production of clotting factors, coupled with immobilization and pre-existing comorbidities which can significantly contribute to VTE [69]. Although a similar, albeit less potent inflammatory response is noted in patients with influenza, intravascular complications are seldom reported in patients with influenza, with only a few isolated cases of severe H1N1 reporting VTE in critically ill patients [70–72]. This suggests that vascular thrombosis is an integral part of COVID-19 disease unlike influenza.

The increased severity of vascular complications in COVID-19 can also be inferred by Merkler et al. recent study comparing rate of ischemic stroke between patients with COVID-19 and patients with influenza. After adjustment for covariates, the likelihood of stroke in patients presenting with COVID-19 was 7.5 times higher than patients with influenza (OR 7.5, 95% CI [2.3–24.9]) [11]. Although the pathophysiology remains less well-defined, immune dysregulation and cytokine storm coupled with an attenuated immune response appear to be key components of progression of COVID-19 to critical disease [73]. Timely pharmacologic VTE prophylaxis with low-molecular weight heparin or unfractionated heparin of high-risk patients is shown to improve outcomes [74]. However, further data is required to evaluate the optimal therapeutic dose of these regimens, given that some studies report VTE events despite standard-dose thromboprophylaxis in patients with COVID-19 [74].

### Established cardiovascular disease and its association with prior influenza or COVID-19

Several studies have evaluated the potential of influenza to trigger cardiovascular events [75–77] owing to the underlying mechanism of systemic inflammation which may cause thrombosis after an acute infection [78]. A meta-analysis by Warren-Gash et al. [75] demonstrated almost a twofold increase in risk of MI after influenza like illness symptoms (OR 2.17, 95% CI [1.68–2.80]; 6658 participants). This association was less evident with serologically defined influenza (OR 1.27, 95% CI [0.54–2.95]; 956 participants); however, the only study which adjusted for confounding variables showed a significant risk of MI after positive influenza serological test (OR 5.50, 95% CI [1.31–23.13]) [75]. Similar association has been observed in other studies as well [43] (Additional file 1: Table S2). For the one study evaluating risk of HF with influenza, three of the seven patients who developed HF were positive for recent influenza [79].

Two case-cross over studies [80, 81] evaluating association between stroke and preceding hospitalization due to influenza-like illness symptoms concluded increased odds of ischemic stroke in the first 15 days post influenza-like illness (OR 2.88, 95% CI [1.86–4.47] [80]; OR 1.39, 95% CI [1.09–1.77] [81]). Boehme et al. [80] reported a decrease in the strength of this relationship as days from influenza-like illness increased as opposed to Alvord et al. [81] where the association increased over time.

Currently, no data exist evaluating the chronic cardiovascular effects of SARS-CoV-2 infection months after recovery from acute illness. One study evaluating myocardial injury in patients recently recovered from COVID-19 yielded 78% patients to have residual cardiac involvement, a finding unlikely to be observed in patients after influenza virus, where transient myocardial injury is more commonly associated with acute viremic phase [82]. Given the similar pathophysiology between the two viruses, whereby an aggravation of systemic inflammatory cytokines lead to cytokine-storm syndrome, a phenomenon commonly responsible for severe COVID-19 [83], induction of new cardiac pathologies or exacerbation of pre-existing CVD is likely [6]. High inflammatory burden can induce vascular inflammation rapidly, which can promote development of atherosclerosis, cardiac arrhythmia, and myocarditis [6]. A much higher burden of cardiovascular complications post COVID-19 compared with influenza can therefore be anticipated, owing to the more potent underlying inflammatory response of SARS-CoV-2.

### Cardiovascular hospitalizations during influenza vs COVID-19 season

Peak influenza season has been associated with increased cardiovascular hospitalizations [84–86]. In a study by Kytomaa et al. [86], community surveillance data used to analyze frequency of MI and heart failure hospitalizations and its association with monthly influenza activity revealed a 5% monthly increase in influenza activity to be associated with a 24% increase in HF hospitalizations (incidence rate ratio [IRR], 1.24; 95% CI, 1.11–1.38; *P* < .001) [86]. Conflicting results exist regarding MI hospitalization [85, 86]. A time-series analysis by Warren-Gash et al. [85] demonstrated a significant association between MI hospitalization and influenza-like illnesses’ consultations in England (IRR for a lag of − 1 week, 1.009 [95% CI, 1.003–1.015; *P* = .004] and proportion of influenza-positive specimens in Hong Kong (IRR for same week, 1.066; 95% CI, 1.024–1.109; *P* = .002); however, no significant association was observed in the study by Kytomaa et al. [86] (Additional file 1: Table S3).

These trends contrast with that observed during the current COVID-19 pandemic, where a decrease of ~ 50% in heart failure hospitalizations has been observed since the first diagnosed case of COVID-19 [87]. Similar downward trajectory has been observed across acute cardiovascular hospitalizations, where a significant decline in daily hospitalization rate was observed throughout March 2020 (− 5.9% per day [− 7.6 to − 4.3%], *P* < 0.001) [88]. These reductions were observed despite a significantly large increase in mortality (up to 90%) due to CVD during this time period, with a transient two-time increase in the incidence of out of hospital cardiac arrests [89, 90]. This signifies that a decrease in hospitalizations may largely be attributed to patient fear of seeking healthcare in a medical facility due to concerns of contracting the virus, which are further substantiated by increased inculcation of physical distancing and isolation. The consequences of avoiding emergent care are greater than the risks imposed by seeking care in a medical facility, and the detrimental effects of delaying appropriate medical care often lead to poor prognosis in these patients. It is crucial to acknowledge that an increase in out-of-hospital cardiac arrests and sudden death signifies that the reduction of cardiovascular hospitalization was not benign, and can have significant consequences in the future if not timely prevented. These stringent measures of social isolation are not commonly adopted to curb transmission of influenza virus, even during peak influenza season; hence, people may not be hesitant to seek medical care which may explain the opposing trajectories of hospitalization observed in the two diseases.

### Modalities for prevention of influenza and SARS-CoV-2

The most effective and cost benefit prophylaxis of influenza virus is the seasonal influenza vaccine. Although the effectiveness of vaccine may vary among groups, recent studies have shown that influenza vaccine reduces the risk of flu by 40–60% during peak influenza seasons [91]. During the 2017–2018 seasonal influenza, an estimated 3.2 million influenza-associated medical visits, 91,000 influenza-associated hospitalizations, and 5700 influenza-associated deaths were prevented due to adequate vaccination prior to the season [91]. In terms of safety, almost all influenza vaccines on the market are well-tolerated [92, 93] and adverse effects are uncommon and usually mild [94].

In addition to preventing the virus, influenza vaccine has also been implicated in preventing major adverse cardiovascular outcomes [95–98]. In a study by Udell et al. [95], pooled data from six randomized clinical trials indicated influenza vaccine to be associated with a lower risk of composite cardiovascular events (2.9% vs 4.7%; RR, 0.64 [95% CI, 0.48–0.86], *P* = .003) [95]. Considering the link of influenza virus and increased risk of MI, the potential protective effect of influenza vaccine in prevention of adverse cardiovascular outcomes is of paramount clinical importance.

Given the high transmission and reproduction number (R_0_ 2.2) of SARS-CoV-2 coupled with its ability to cause a pandemic disease within weeks, a possibility to curb this virus without a vaccine seems highly challenging. Currently, numerous vaccines to combat COVID-19 are under clinical evaluation while over a hundred vaccines are undergoing pre-clinical evaluation [99]. Developing a novel vaccine which is safe and effective in a short time frame poses its own challenges. One of the obstacles in early development of SARS coronavirus vaccines has been the finding of undesired immunopotentiation which often occurs after immunizations with whole virus or complete spike protein vaccines [28, 100]. Second, there is also heightened concerns regarding the adverse effects these vaccine trials might pose such as exacerbating ongoing lung and cardiovascular diseases [101]. Given that the most vulnerable population such as healthcare workers and elderly will be prioritized for these vaccine trials, it is crucial to accurately determine and review the safety before these subgroups are exposed to it. Third, vaccine development is a lengthy and expensive process due to the high failure rates which raises concerns as to whether the vaccine will be developed in time to combat this pandemic [101]. One way to counter this is by using a “pandemic paradigm,” which supports multiple activities during vaccine development to be executed in parallel before confirming a successful outcome of the another step [101]. For example, human phase 1 clinical trials can proceed parallel to ongoing testing in animal models [101].

Considering that clinical trials for influenza vaccines first began in mid-1930s [102, 103], and the first bivalent vaccine which provided proof of effective protection against flu epidemics became available in December 1942 [104, 105], it is likely that we might be years from developing an effective vaccine to prevent COVID-19. The past research on SARS and Middle East respiratory syndrome (MERS) may help fast-track the development of potential vaccine.

Until a vaccine is available, the most effective barriers in reducing transmission risk of SARS-CoV-2 include physical distancing, hand hygiene, and facemask protection [106]. Strong evidence suggests wearing facemasks can efficaciously reduce virus particles in respiratory droplets. Study by Stutt et al. concluded that the use of routine facemask by up to half of the population can reduce *R* value to less than 1.0, thereby preventing the possibility of another wave [107]. These measures of protection extend well beyond SARS-CoV-2, by also mitigating risk of transmission of influenza virus. Adopting facemask protection at a population level can effectively decrease the influenza infection attack rate (R_int_) beyond the threshold of 1.0, thereby controlling the possibility of an influenza pandemic [108]. Adequate compliance of these measures is therefore crucial in an effort to delay or contain the possibility of a dual epidemic.

Although speculative, if COVID-19 vaccine is as effective as influenza vaccine, it may additionally reduce adverse cardiovascular events owing to the suppression of acute inflammatory and procoagulant stimulus, a mechanism postulated in prevention of alteration in endothelial function and consequent destabilization of vulnerable atherosclerotic plaques which may contribute to coronary artery occlusion in influenza vaccine [109]. This can serve as a possible secondary prevention for individuals with underlying CVD, who are susceptible to recurrent cardiovascular events due to COVID-19 infection.

### Limitations

Although maximum efforts were utilized to collate all available evidence, there are a few limitations that should be considered. First, we only included major observational studies evaluating influenza or COVID-19 concurrent with cardiac abnormality, thereby excluding data available within case-series or case reports. Second, majority of the studies included were retrospective in design, which are subject to bias due to missing information or recall error. Third, since influenza is a self-limiting disease, majority cases often go unreported with only patients with severe illness requiring emergency care as opposed to COVID-19, where majority of those infected were hospitalized. This also signifies the severity of underlying disease. Fourth, studies involving COVID-19 were from China and the USA only, and thus, their findings cannot be generalized to other regions. Fifth, we offer an indirect comparison between COVID-19 and influenza, by including studies evaluating disease progression in different centers during different time period. Final, no studies including patient population co-infected with COVID-19 and influenza were analyzed, which can potentially cause a different trajectory of disease progression.

## Conclusion

**Fig. 2:**
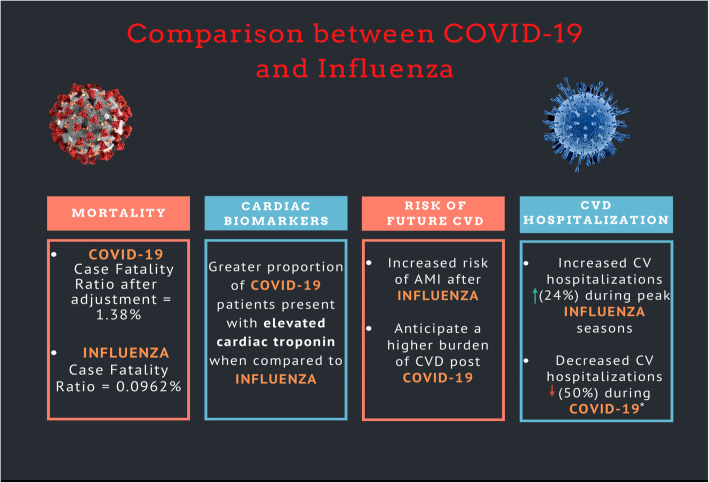
Overall comparison between influenza and COVID-19. *Contrary to influenza, a transient two times increase in out of hospital cardiac arrests was observed during COVID-19 pandemic period, suggesting indirect effects of lockdown and reluctance of patients to present to the hospital out of fear of contamination

This review highlights the similarities and differences between influenza and COVID-19 and the potential risks associated with coincident pandemics (Fig. 2). COVID-19 has a higher mortality and case fatality rate and has increased risk of adverse outcomes especially in patients with underlying CVD. Vascular complications including DVT and PE are markedly higher in COVID-19 patients suggesting that vascular thrombosis is an integral part of the disease unlike influenza. The decreased rates of hospitalization during COVID-19 pandemic further pose a threat to patients who experience diseases which require prompt in-hospital treatment such as myocardial infarction or stroke, where a possible delay in care can lead to permanent impairment. In the coming months increasing efforts towards evaluating new interventions will be vital to curb COVID-19 especially as peak influenza season approaches. It is crucial to acknowledge that the higher number of patients hospitalized due to COVID-19 globally, ranging from moderate to severe disease gives physicians and researchers alike an opportunity to look at relatively rare complications with much more clarity and granularity when compared with acute influenza, where hospitalizations are much less common leading to paucity of data pertaining rare complications of the disease. Given the similar clinical presentation of both viruses, timely detection by accurately distinguishing COVID-19 from influenza pneumonia through imaging can help in early management of these patients. Currently, not enough data exist regarding co-infection of COVID-19 with influenza or how it would progress clinically though it may cause a significant burden on an already struggling health care system. Until an effective COVID-19 vaccination is available, high coverage of influenza vaccination should be of utmost priority to ensure patient safety and prevent the possibility of a co-epidemic [110], which poses as a major health threat to non-infected patients with underlying CVD. Government, hospital administrators, and policy makers should work together to prepare for a substantial increase in health care resources, and multi-national collaborations should be encouraged to advance high quality research to combat this pandemic and its associated downstream cardiovascular and other health complications.

## Supplementary Information

**Additional file 1: Table S1A.** [Cardiac biomarkers evaluating risk of myocardial injury in patients presenting with acute influenza]. **Table S1B.** [Cardiac biomarkers evaluating risk of myocardial injury in patients presenting with COVID-19]. **Table S2.** [Studies highlighting Chronic Cardiovascular Complication and its association with prior Influenza]. **Table S3.** [Studies evaluating association between Influenza season and hospitalization for cardiovascular conditions].

## Data Availability

All data generated or analyzed during this study are included in this published article [and its supplementary information files].

## Abbreviations

ARDS: Acute respiratory distress syndrome
CDC: Centers for Disease Control and Prevention
CK: Creatine kinase
COVID-19: Coronavirus disease 2019
CVD: Cardiovascular disease
hs-cTnT: High-sensitivity cardiac troponin T
ICU: Intensive care unit
MERS: Middle East respiratory syndrome
MI: Myocardial infarction
PE: Pulmonary embolism
SARS-CoV-2: Severe acute respiratory syndrome coronavirus 2
VTE: Venous thromboembolism
