# Supplementary material for: Cardiovascular implications of COVID-19 versus influenza infection: a review
Source: BMC Med. 2020 Dec 18;18:403. doi: 10.1186/s12916-020-01816-2 (PMC7746485; doi:10.1186/s12916-020-01816-2)
Supplement: Supplementary file 1 — Additional file 1: Table S1A. [Cardiac biomarkers evaluating risk of myocardial injury in patients presenting with acute influenza]. Table S1B. [Cardiac biomarkers evaluating risk of myocardial injury in patients presenting with COVID-19]. Table S2. [Studies highlighting Chronic Cardiovascular Complication and its association with prior Influenza]. Table S3. [Studies evaluating association between Influenza season and hospitalization for cardiovascular conditions]. [file 12916_2020_1816_MOESM1_ESM.docx]

**Additional File 1**

**Table S1A: Cardiac biomarkers evaluating risk of myocardial injury in patients presenting with acute influenza**

**Table S1B: Cardiac biomarkers evaluating risk of myocardial injury in patients presenting with COVID-19**

**Table S2: Studies highlighting Chronic Cardiovascular Complication and its association with prior Influenza**

**Table S3: Studies evaluating association between Influenza season and hospitalization for cardiovascular conditions**

**Table S1A: Cardiac biomarkers evaluating risk of myocardial injury in patients presenting with acute influenza**

| **Study** | **Country** | **Study Design** | **N** | **CVD, (%)** | **D-dimer** | **CRP** | **Troponin** | **Troponin T** | **Troponin I** | **CK** | **CK-MB** | **NTproBNP** |
| --- | --- | --- | --- | --- | --- | --- | --- | --- | --- | --- | --- | --- |
| Greaves, 2003 | USA | Randomized placebo-control trial | 152 | NR | NR | NR | NR | NR | NR | 12 patients had high CK [1064 (181 - 7280)] | elevated 21 (8-80) | NR |
| Ison 2005 | USA | Prospective | 30 | NR | NR | NR | None had elevated troponin | NR | NR | 2 patients had high CK [312  (37-5430)] | NR | NR |
| Cao, 2009 | China | Retrospective | 426 | 2 (0.5) | NR | CRP >10 [mg/L] in  68/217 patients | NR | NR | NR | CK >200 [U/L]  In 16/115 patients | NR | NR |
| Erden, 2010 | Turkey | Prospective | 28 | 0 | NR | NR | NR | NR | NR | 6 patients had elevated CK | 17.9 [IU/L] | NR |
| Wang, 2011 | China | Retrospective | 92 | NR | Non- Fatal group 5.75± 4.11 [ug/mL]  Fatal group 10.70± 6.29  [ug/mL] |  |  |  |  | Non- Fatal group 591.54± 348.72  [U/L]  Fatal group 682.20± 424.20  [U/L] | Non- Fatal group 30.30± 17.63  [U/L]  Fatal group 32.66± 18.25 [U/L] |  |
| Chacko, 2012 | India | Retrospective | 37 | 5.4 | NR | NR | NR | NR | 17 patients with >1.5 [ng/mL]  3.1 (7.7) [ng/mL] | NR | 34 patients 106 ± 137 [U/L] | NR |
| Fagnoul, 2013 | Belgium | Retrospective | 46 | 10.9 | NR | NR | 0.03 (0.01-0.3)  [ng/mL] | NR | NR | NR | NR | NR |
| Han, 2015 | China | Retrospective | 40 | 6 (0.15) | NR | NR | NR | NR | Group II  0.007 (0.oo-0.032)  [pg/mL]  Group III  0.388 (0.003-1.449)  [pg/mL]  Group IV  1.145 (0.073-3.024)  [pg/mL] | Group II  218 (38-1048)  [U/L]  Group III  493  53–2622  [U/L]  Group IV  440  90–889  [U/L] | Group II  22 (11-62)  [U/L]  Group III  33 (15-79) [U/L]  Group IV  28 (25-34) [U/L] | NR |
| Ito, 2017 | Japan | Prospective | 102 | 0 | NR | NR | None of the patients had elevated troponin | NR | NR | NR | 5 patients had elevated CK-MB  17 (16-36) [IU/mL] | NR |
| Harris, 2018 | USA | Retrospective | 33 | 42.4 | NR | NR | NR | NR | 23 patients with 0.3 to 1.5 [ng/mL]  10 patients with > 1.5 [ng/mL]. | NR | NR | NR |
| Pizzini, 2020 | Austria | Cross-sectional analysis | 264 | 33.7 | NR | IV-A 4.74 ± 5.89 [mg/dL]  IV-B  0.98 ± 0.30 [mg/dL] | NR | hsTnT  IV-A  27.09 ± 67.31 [ng/L]  IV-B  16.40 ± 36.3 [ng/L] | NR | NR | NR | IV-A  3238 ±7992  [ng/L]  IV-B  1716 ± 2572  [ng/L] |
| Gao, 2020 | China | Retrospective | 321 | 8.1 | NR | NR | NR | NR | 0.5 (0.2–2.1)  Maximum level was 1.1 (0.3–5.1) | NR | 0.9 (0.5–1.5)  Maximum level was 1.3 (0.8–2.4) | NR |

CRP = C-Reactive Protein; CK = Creatine Kinase; CK-MB = Creatine Kinase-myocardial band; NR = Not Reported; hsTnT = high sensitivity cardiac troponin T; IV = Influenza virus; CVD = cardiovascular disease; NTproBNP = N-terminal pro b-type natriuretic peptide
Data reported as N (%), mean (SD), median (IQR)

**Table S1B: Cardiac biomarkers evaluating risk of myocardial injury in patients presenting with COVID-19**

| **Study** | **Country** | **Design** | **N** | **CVD (%)** | **CRP** | **D-Dimer** | **Troponin** | **Troponin T** | **Troponin I** | **CK** | **CK-MB** | **NTproBNP** | **BNP** |
| --- | --- | --- | --- | --- | --- | --- | --- | --- | --- | --- | --- | --- | --- |
| Cummings, 2020 | US | Prospective | 257 | 19.1 | 158 (92–254)/ 253  [mg/L] | 1·6 (0·9–3·5) in 244 patients  [ug/mL] | NR | HsTnT  19 (9–52) in 254 patients  [ng/L] | NR | NR | NR | NR | NR |
| Richardson, 2020 | US | Retrospective case-series | 5700 | 12.3 | 13.0 (6.4-26.9) mg/dL | 438 (262-872) ng/mL | Elevated troponin in 802 patients | NR | NR | 171 (84-397)  [U/L] | NR | NR | 385.5 (106-1996.8)  [pg/mL] |
| Arentz, 2020 | US | Retrospective case-series | 21 | 42.9 | NR | NR | 3 patients with Troponin > 0.3 [ng/mL] | NR | NR | 95 (45-1290) U/L | NR | NR | 4720 (69-33423) pg/mL |
| Chen N, 2020 | China | Retrospective | 99 | 40.4 | 51.4 (41.8) [mg/L]  Increased in 63/73 patients | 0.9 (0.5-2.8)  [ug/L]  Increased in 36 patients |  |  |  | 85.0 (51.0 -184.0) [U/L]  Increased in 13 patients |  |  |  |
| Zhou, 2020 | China | Retrospective | 41 | 14.6 | NR | 0·8 (0·4–3·2)  [ug/L] | NR | NR | hsTnI  4·1 (2·0–14·1)  [ng/mL] | 21·5 (13·0–72·4)  [U/L] | NR | NR | NR |
| Huang, 2020 | China | Cohort | 41 | 14.6 | NR | 0·5 (0·3–1·3)  [mg/L] | NR | NR | hsTnI  3·4 (1·1–9·1)  [pg/mL] | 132·5 (62·0–219·0) | NR | NR | NR |
| Guan, 2020 | China | Retrospective | 1099 | 2.5 | >10mg/L in 481/793 patients | >0.5 mg/L in 260/560 patients | NR | NR |  | CK >200 [U/L] in 90/657 | NR | NR | NR |
| Guo, 2020 | China | Retrospective | 187 | 15.5 | 4.04 (1.64-8.14) [mg/dL] | 0.43 (0.19-2.66) [ug/mL] |  |  |  |  |  | 268.4 (75.3-689.1) [pg/mL] |  |
| Wang, 2020 | China | Retrospective case-series | 138 | 14.5 | NR | 203 (121-403) [mg/dL] | NR | NR | hsTnI  6.4 (2.8-18.5) [pg/mL] | 92 (56-130) [U/L] | 14 (10-18) [U/L] | NR | NR |
| Zhang, 2020 | China | Retrospective | 138 | NR | 34.2 (12.5-67.4)  [Mg/L] in 125/136 patients | 0.20 (0.1-0.5)  [ug/mL]    Increased in 35/81 patients | NR | NR | NR | 72.5 (52.2-115)  [U/L] in 4/60 patients | NR | NR | NR |
| Yang, 2020 | China | Retrospective | 52 | 9.6 | NR | NR | NR | NR | hsTnI 161(41·8–766·1) [pg/mL] | NR | NR | NR | NR |
| Wu, 2020 | China | Cohort | 201 | 4.0 | 42.4 (14.2-92.7) [mg/L]  in  194 patients | 0.61 (0.35-1.28) [ug/mL] in 189 patients | NR | NR | NR | NR | 15 (12-20) U/L in 198 pts | NR | NR |
| Chen T, 2020 | China | Retrospective | 274 | 8.4 | 53.4 (18.6-113.0) [mg/L] | 1.1 (0.5-3.2) [μg/mL] |  |  | hsTnI 8.7 (2.9-33.6) [pg/mL] | 109.0 (53.5- 188.0) [U/L] |  | 267.0 (48.0-821.0) [pg/mL] |  |
| Lang Wang, 2020 | China | Retrospective | 339 | 15.6 | 49.6 (18.5-93.2) [mg/L] | 1.20 (0.62-3.25) [mg/L] | NR | NR | hsTnI 0.010 (0.006-0.030) [ng/mL] | 63 (40-104) [U/L] | 1.26 (0.85-2.36) [ng/mL] | NR | NR |
| Shi, 2020 | China | Cohort | 416 | 10.6 | 4.5 (1.4-8.5) [mg/dL] |  | NR | NR | hsTnI <0.006 (<0.006-0.02) [ug/L] | NR | 1.0 (0.7-2) [ng/mL] | 219 (73-699)  pg/mL | NR |
| **Summary** | | | | | | | | | | | | | |
| Less heterogeneous data is observed for cardiac biomarkers evaluating the risk of myocardial injury in patients presenting with COVID-19. Fourteen studies evaluated at least two or more cardiac biomarkers, with CRP, D-dimer, HsTnI and CK being the most commonly reported cardiac biomarkers. D-dimer values were reported in 12 studies, while NT-proBNP and BNP levels were reported in three and two studies respectively. | | | | | | | | | | | | | |

Levels noted at the time of hospitalization
CRP = C-Reactive Protein; CK = Creatine Kinase; CK-MB = Creatine Kinase-myocardial band; hsTnI = hypersensitive cardiac troponin I; NR = Not Reported; hsTnT = high sensitivity cardiac troponin T; CVD = Cardiovascular Disease; NTproBNP = N-terminal pro b-type natriuretic peptide

Data reported as mean (SD), N(%) or median (IQR)

**Table S2: Studies highlighting Chronic Cardiovascular Complication and its association with prior Influenza**

| **Study Author, Year** | **Study Design** | **Country** | **Influenza confirmation** | **Outcome of Interest** | **(N)** | **Influenza**  **Positive** | **Cases** | **Controls** | **Results** |
| --- | --- | --- | --- | --- | --- | --- | --- | --- | --- |
| Guan, 2008 | Case-control | China | Serology | AMI | 158 | IV-A: 122 IV-B: 73 | 78 | 80 | IV-A OR 7.5 (1.3–43.0) IV-B OR 27.3 (6.6–113.8)  IV-B had closest association with the risk of AMI than that of other pathogens (OR 27.3 [6.6 -113.8], P<0.001). |
| Kwok et al, 2015 | Meta-analysis of 12 studies | NA | Both | MI, HF, stroke | 84,003 | NA | NA | NA | Serological Influenza: MI (OR 1.27 [95% CI, 0.54–2.95]; ILI: MI (OR 2.17 95% CI [1.68–2.80])  HF: 1 study. Crude OR for risk of HF (OR 7.05 95% CI [1.22– 40.90])  Stroke: 1 study. Increased risk of stroke within first 15 days of ILI (OR 6.5, 95% CI [2.2–19.7]) which decreases with duration (OR 3.3 95% CI [1.9–5.8]) at 90 days. |
| Warren-Gash, 2018 | Self-controlled case-series design | Scotland | Serology | MI, Stroke | 1989 | 293 | 1989 | None | MI: Age and season-adjusted IR (95% CI):  Day 1-3: 9.80 (2.37-40.5); Day 4-7: 3.98 (0.55-28.9); Day 8-14 2.72 (0.38-19.5); Day 15-28: 2.77 (0.68-11.2); vs all other respiratory viruses (combined)  Stroke: Age and season-adjusted IR (95% CI): Day 1-3, 7.82 (1.07-56.9); Day 8-14, 8.13 (1.98-33.3); Day 15-28, 5.13 (1.55-17.0) vs all other respiratory viruses (combined) |
| Boehme AK, 2018 | Case‐crossover design | US | ILI | Preceding ILI in stroke patients | 36975 | 554 | 36975 | Cases without past influenza act as control | ILI within 15 days before stroke OR 2.88 (1.86–4.47); 30 days before stroke OR 2.59(1.86–3.61); 60 days before stroke OR 2.18(1.69–2.82; 90 days before stroke 1.78 (1.43–2.21); 180 days before stroke 1.51(1.27–1.78; 365 days before stroke 1.50(1.31–1.71). |
| Kwong, 2018 | Self-controlled case-series design | Canada | Serology | AMI | 332 | 332 |  |  | Risk Interval for AMI after influenza infection: 1-7 days IR 6.05 (3.86–9.50); 1-3 days IR 6.30 (3.25–12.22); 4-7 days IR 5.78 (3.17–10.53); 8-14 days IR 0.60 (0.15–2.41); 15-28 days IR 0.75 (0.31–1.81) |
| Alvord, 2019 | Abstract: case-crossover | US | ILI | Preceding ILI in stroke patients | 30912 | NR | 30912 | None | ILI within 15 days before stroke OR 1.39 (1.09–1.77); 30 days before stroke OR 1.48 (1.25–1.76); 60 days before stroke OR 1.48 (1.30–1.69); 90 days before stroke 1.59 (1.42–1.78); 180 days before stroke 1.68(1.53–1.85); 365 days before stroke 1.69(1.56–1.83) |
| Ohland, 2020 | Self-controlled case series | Denmark | Serology | MI, stroke | 1350 | NR | 1350 | None | MI Season adjusted IR: Day 1-3, 17.5 (8.5–36.2); Day 4-7, 5.1 (1.6–16.3); Day 8-14, 3.1 (1.0–9.7); Day 15-28, 1.7 (0.5–5.3)  Stroke Season adjusted IR: Day 1-3, 10.3 (4.2–25.4); Day 4-7, 6.5 (2.4–17.7); Day 8-1, 5.9 (2.6–13.4); Day 15-28, 1.0 (0.3–4.2) |

ILI = Influenza-like illness; HF = Heart Failure; MI = Myocardial Infarction; AMI = acute myocardial infarction; IV = Influenza Virus OR = Odds ratio; RR = Risk Ratio; IR = Incident Ratio

**Table S3: Studies evaluating association between Influenza season and hospitalization for cardiovascular conditions**

| **Study Author, Year** | **CV Area** | **Objective** | **Outcome** |
| --- | --- | --- | --- |
| Upshur, 1999 | CHF | Relation between circulating influenza virus and all hospital admissions of people >65 years | The relation between circulation of influenza virus and admissions for congestive heart failure was inconsistent. |
| Warren-Gash, 2011 | MI | Relationship between population levels of influenza circulation and acute MI–associated admissions and deaths in England and Wales and in Hong Kong | (IRR, 1.05 [1.043–1.058]; P < .001)*  (IRR, 1.08 [1.013–1.145]; P = .018) ‡ |
| Foster, 2013 | AMI, Stroke | To determine if AMIs and ischemic strokes are associated with influenza activity | Across all models, we found consistent significant associations between AMIs and influenza activity, but not between ischemic strokes and influenza. |
| Kytomaa, 2019 | HF, MI | To estimate annual influenza-associated hospitalizations in the USA by hospital discharge category, discharge type, and age group. | (IRR, 1.24 [1.11-1.38]; *P* < .001) ~  (IRR, 1.02 [0.90-1.17]; p= 0.72) # |

HF = Heart Failure; MI = Myocardial Infarction; AMI = acute myocardial infarction; CHF = Congestive Heart Failure; IRR = Incident rate ratio
~ A 5% monthly absolute increase in influenza activity was associated with a 24% increase in HF hospitalization rates
# Influenza-like illness activity was not significantly associated with MI hospitalizations

*Association in England and Wales between General practitioner consultations for Influenza like illness and MI-associated deaths after adjusting for environmental temperature and humidity
‡ Significant association between the proportion of specimens testing positive for influenza virus and MI-associated deaths occurring in the same week in Hongkong
